# Structural and Microvascular Changes in the Macular Are Associated With Severity of White Matter Lesions

**DOI:** 10.3389/fneur.2020.00521

**Published:** 2020-06-30

**Authors:** Chenlei Peng, William Robert Kwapong, Shasha Xu, Farah Mohamed Muse, Jueyan Yan, Man Qu, Yungang Cao, Hanpei Miao, Zhenxiang Zhen, Bo Wu, Zhao Han

**Affiliations:** ^1^The Second Affiliated Hospital and Yuying Children's Hospital of Wenzhou Medical University, Wenzhou, China; ^2^West China Hospital, Sichuan University, Sichuan, China; ^3^Taizhou Central Hospital, Taizhou University Hospital, Zhejiang, China; ^4^School of Ophthalmology and Optometry, Wenzhou Medical University, Wenzhou, China

**Keywords:** white matter hyperintensity, macula, Fazekas scale, optical coherence tomographic angiography, magnetic resonance imaging

## Abstract

**Purpose:** This study aimed to characterize the microvascular and structural changes in the macular that occur in white matter hyperintensities (WMH) using optical coherence tomographic angiography. We also aimed to explore the association between macular microvascular and structural changes with focal markers of brain tissue on MRI in WMH using the Fazekas scale.

**Methods:** This study enrolled healthy participants who were stroke- and dementia-free. MRI was used to image the cerebral white matter lesions, and Fazekas scale was used to evaluate the severity of the white matter lesions. Optical coherence tomography angiography (OCT-A) was used to image the radial peripapillary capillaries (RPCs), macular capillary plexuses [superficial capillary plexus (SCP) and deep capillary plexus (DCP)] and thickness around the optic nerve head, peripapillary retinal nerve fiber layer (pRNFL).

**Results:** Seventy-four participants were enrolled and divided into two groups according to their Fazekas score (Fazekas scores ≤ 1 and ≥2). Participants with Fazekas score ≥2 showed significantly reduced RPC density (*P* = 0.02) and DCP density (*P* = 0.012) when compared with participants with Fazekas score ≤ 1. Participants with Fazekas score ≥2 showed reduced pRNFL (*P* = 0.004) when compared to participants with Fazekas score ≤ 1. Fazekas scores were significantly associated with the pRNFL thickness (Rho = −0.389, *P* = 0.001), RPC density (Rho = −0.248, *P* = 0.035), and DCP density (Rho = −0.283, *P* = 0.015), respectively.

**Conclusions:** Microvascular impairment and neuro-axonal damage are associated with the disease cascade in WMH. We have shown that RPC and DCP densities are significantly affected, and these impairments are associated with the severity of the disease and cognitive function. OCT-A could be a useful tool in quantifying the retinal capillary densities in WMH.

## Introduction

With the rise in the aging population, cerebral neurodegenerative diseases such as dementia and Alzheimer's disease have been reported to be more common the elderly (1, 2). As such, researchers and clinicians are working to preserve a high quality of life in the aging population and also prevent the occurrence of these diseases (3). Due to the irreversibility of dementia and the insufficient therapy other than therapy to counteract the symptoms, current research studies are more focused on the earliest or preclinical stages to deter the disease progression and to improve treatment outcomes. Detection of dementia and other neurodegenerative diseases associated with aging before its clinical manifestation will be key for its prevention; however, there are insufficient simple and reliable imaging modalities to screen for asymptomatic subjects at risk of developing dementia or other cerebral neurodegenerative diseases.

White matter hyperintensities (WMHs) are lesions that show up as areas of increased brightness when visualized by T2-weighted magnetic resonance imaging (MRI); WMH has been reported to be the unavoidable substrate for the development in most cerebral small vessel diseases (cSVDs) such as Alzheimer's disease and dementia (4). Individuals with a higher burden of WMH are at high risk of developing dementia (5) and stroke (6). WMH has been reported to be as a result of a chronic ischemic injury caused by small vessel disease in the brain (7). Screening tools such as magnetic resonance imaging (MRI) and positron emission tomography (PET) have shown potential in detecting these white matter changes in the brain but are often limited by the expense, long time involved in imaging, and its limited use on participants (such as cannot be used on participants with stents in the heart); additionally, these imaging modalities detect these changes at the later phase of the disease. As such, researchers and clinicians are interested in structural biomarkers for the early detection of WMH so as to help curb or deter its progression.

Accumulating evidence has shown that the retina vasculature could be a potential medium for analyzing the changes that occur in the cerebral vasculature because the retina shares many histological, physiological, and embryological features with the brain (8, 9). Subtle neuronal and vascular alterations in the retina reflect the pathological vascular changes of the brain during a disease cascade such as Alzheimer's disease (10). Reports using optical coherence tomographic angiography (OCT-A) have been applied to neurodegenerative diseases such as dementia (11) and stroke (12), which are normally associated with distinct microvascular changes and also are associated with the later phase of WMH. These reports are important because they suggest that the OCT-A can be a potential biomarker in these neurodegenerative diseases and may help elucidate the possible mechanism(s) involved in WMH. The aim of our current study is to characterize the structural and microvascular changes in the macular that occur in WMH using the OCT-A; we also aimed to explore the macular structural and microvascular changes with focal markers of brain tissue on MRI in WMH using the Fazekas scale.

## Methods

Eighty participants who were stroke- and dementia-free were recruited from the Second Affiliated Hospital and Yuying Children's Hospital of Wenzhou Medical University, China. This was done from January 2019 to October 2019 on individuals who had their annual medical checkups at the hospital. The inclusion criteria for our study were as follows: (1) aged ≥60 years, (2) Chinese mandarin speaking, (3) sufficient sensorimotor and language competency for cognitive testing, and (4) provided written informed consent. The exclusion criteria were as follows: (1) history of clinical stroke or transient ischemic attack ascertained by medical records; (2) history of neurological or psychiatric conditions affecting cognition; (3) dementia determined by medical history; (4) evidence of brain tumors, cerebral infarcts larger than 20 mm in diameter or hydrocephalus on MRI; and (5) subjects with medial temporal lobe atrophy (MTA) as defined by a rating of >2 rated on coronal images on T1-weighted brain MRI using the Schelten's 5-point (0–4) scale to exclude prodromal Alzheimer's disease. Added exclusion criteria for retinal-image acquisition include (6) patients with known retinal disease or disease influencing vessel structure in color retina images, such as mild diabetic retinopathy, age-related maculopathy, central serous chorioretinopathy, postcataract extraction, and retinal pigment epithelial detachment (*n* = 2).

Other exclusion criteria included (1) white matter hyperintensity caused by multiple sclerosis or infectious, metabolic, toxic, or metastatic disease; (2) history of hemorrhagic stroke, brain tumors, or cerebral hemorrhage; (3) associated systemic disorders that potentially affect the optic fundus, such as severe liver disease, kidney or heart failure, severe infection, malignant disease, systemic lupus erythematosus, or hereditary disease; (4) local eye disorders that could cause optic fundus disease, such as various eye inflammatory responses or eye surgeries such as cataract extraction or laser surgery within 6 months prior to admission, hypertensive retinopathy, retinal vascular disease; (5) disturbance of consciousness; or (6) high refractive error (± 6.00 D spherical equivalent). The healthy controls, who were without diabetes and well-controlled hypertension, underwent neurological examinations to rule out neurological diseases.

Collection of information on vascular risk factors such as hypertension, diabetes mellitus, hyperlipidemia, and heart disease was done. Hypertension was defined as systolic blood pressure of ≥140 mmHg or diastolic blood pressure of ≥ 90 mmHg or current treatment with antihypertensive medications. Diabetes mellitus was defined as fasting plasma glucose ≥ 6.1 mmol/L or HbA1c ≥ 5.8% or current treatment with blood glucose-lowering medication. Hyperlipidemia was diagnosed according to established guidelines or current treatment with statin medications. Heart disease was defined as history or current cardiac arrhythmia, atrial fibrillation, left ventricular hypertrophy, congestive heart failure, ischemic heart disease, myocardial infarction, electrocardiographic abnormalities, or possible cardiac embolic source. Trained neurologists performed cognitive assessment [Mini-Mental State Examination (MMSE)] on all participants. Clinical data and examination, ocular examination, and MRI assessments were done within a month. Informed written consent was obtained from each subject who participated in the study, and the study was in accordance with the Declaration of Helsinki and was approved by the Ethics Committee of the Second Affiliated Hospital and Yuying Children's Hospital of Wenzhou Medical University.

### Cerebral Structural MRI

All eligible participants underwent MRI scans equipped with 3.0 T superconducting magnets (Signa HDxt GE healthcare) [T1-weighted, T2-weighted, diffusion-weighted imaging, fluid-attenuated inversion recovery (FLAIR)], susceptibility-weighted imaging (SWI) axial sequences, and T1-weighted sagittal sequences. Axial images were angled to be parallel to the anterior commissure-posterior commissure line. Trained and certified radiologists, who were incognito to the participants' clinical condition and retinal imaging findings, assessed the digitized scan data on a personal display workstation at the MRI reading center. When evaluating for WMHs, focal abnormalities were ignored; therefore, if a side or both sides of the brain were focally abnormal, estimates were based on the uninvolved areas. The spin density images (repetition time of 3,000 ms; echo time of 30 s) were used to estimate the overall volume of periventricular and subcortical white matter signal abnormality. Slice thickness was 6 mm, with an interslice gap of 20%.

### Visual Scoring of WMH Burden

Visual rating scales of Fazekas were applied on FLAIR images (range, 0–3) as previously reported (13). Participants were then divided into two groups according to their Fazekas score (Fazekas scores ≤ 1 and ≥2). Ratings were done by a specialist who was masked to each participant's clinical information.

### Retinal Photography

Stereo photographs of the retina and optic disks were taken of each participant in this study using the fundus camera (Zeiss VISUCAM Fundus Camera 224). Trained ophthalmologists at the Eye Hospital of Wenzhou Medical University, who were masked to the participants' characteristics, evaluated the fundus images for the presence of abnormalities in the macula and optic disk. Abnormalities of the macular and optic disk were defined as present if any of the following lesions were detected: retinal hemorrhages (*n* = 2), soft and hard exudates, macular edema, optic disk swelling, and microaneurysms and excluded.

### Basic Ophthalmic Examination

All participants underwent a slit lamp and ophthalmoscopy examination to exclude potential eye diseases. Intraocular pressure (IOP) was measured using a full Auto Tonometer (TX-F; Topcon, Tokyo, Japan), and axial length was measured using Lenstar (Haag-Streit AG, Koeniz, Switzerland) in all participants. The best-corrected visual acuity (BCVA) was examined using a Snellen chart; both eyes of each participant were measured.

### Spectral-Domain Oct

#### pRNFL Thickness

The Avanti RTVue-XR (Optovue, Fremont, California, USA; software V.2017.100.0.1) with a tuning range of 100 nm was used in spectral-domain OCT. The image resolution of each OCT image was 5.3 mm axially and 18 mm laterally. The RNFL thickness was obtained using the optic nerve map protocol, with a scanning range covering a circle with a diameter of 3.45 mm on the optic disk. For the inclusion criteria in our present study, high-quality images with signal strength index ≥6 were accepted according to the OSCAR-IB criteria (14).

#### Spectral-Domain OCT Angiography

OCT angiography images were obtained using Avanti RTVue-XR, which is based on split-spectrum amplitude decorrelation algorithm. Radial peripapillary capillary (RPC) network was obtained in scans within a 0.7-mm wide elliptical annular region extending outward from the optic disk boundary, and the vasculature within the internal limiting membrane and the nerve fiber layer were analyzed automatically using the OCT-A software. The images consisted of two sets of B scans repeated horizontally and vertically, each consisting of 400 A scans.

**Figure 1:**
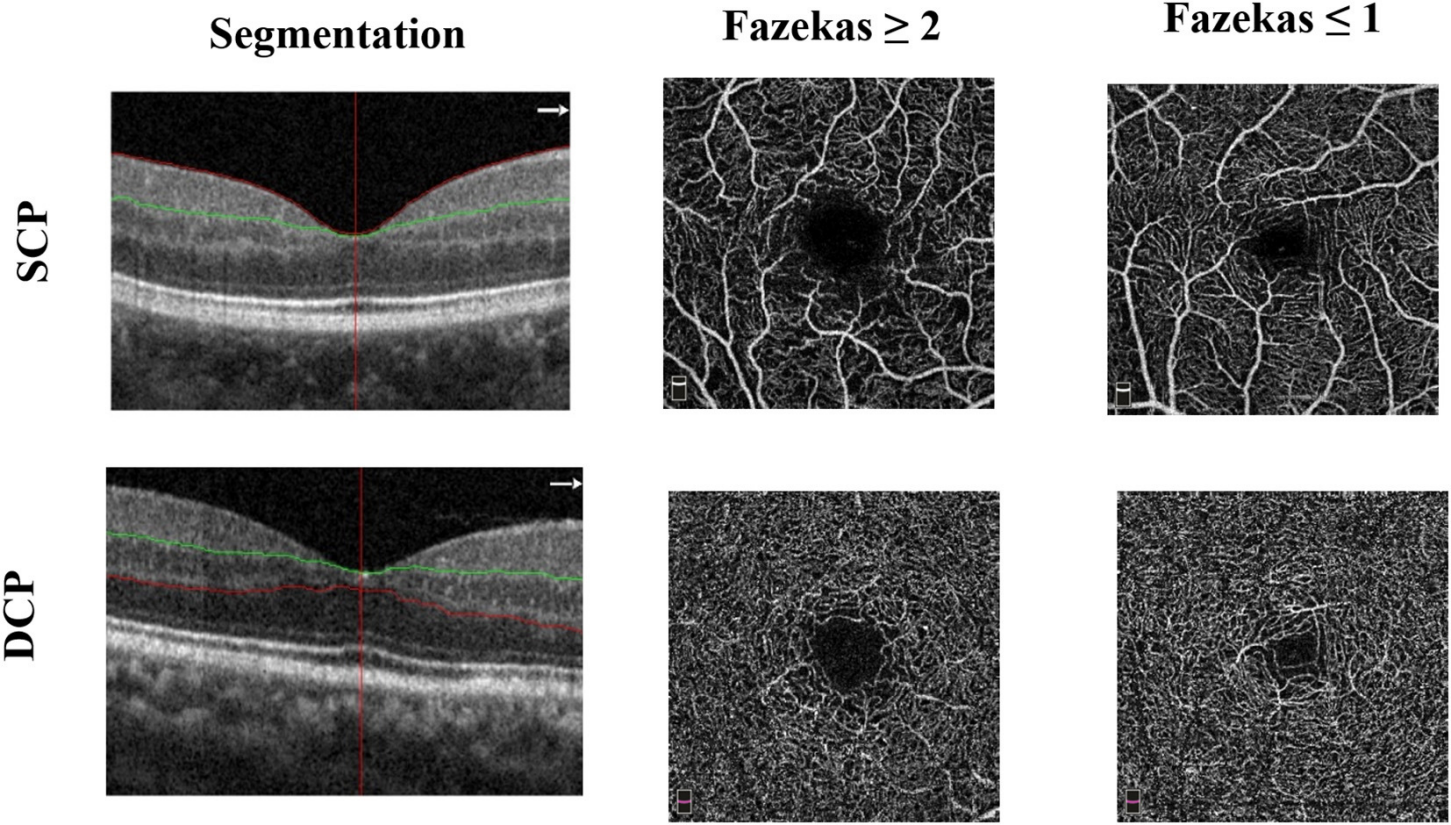
Representative optical coherence tomography angiography (OCT-A) images of the 3 × 3 mm^2^ en face images of the superficial (SCP) and deep capillary plexus (DCP) among the two groups.

The superficial and deep retinal capillary plexuses were detected and separated automatically using the OCT tool. The superficial retinal capillary plexus (SCP) extended from 3 μm below the internal limiting membrane to 15 μm below the inner plexiform layer; the deep retinal capillary plexus (DCP) extended from 15 to 70 μm below the inner plexiform layer (IPL) (Figure 1). A parafoveal capillary network was acquired through 3 × 3 mm^2^ scans within the annular zone of 0.6–2.5 mm diameter around the foveal center. Vessel densities, defined as the percentage area occupied by the large vessels and microvasculature in the analyzed region, were automatically generated in the whole scan area and in all sections of the applied grid according to the Early Treatment Diabetic Retinopathy Study (ETDRS). The quality of OCT angiography images was evaluated by three independent ophthalmologists blinded to the participant's clinical information. Poor quality images with a signal strength index <6 or with residual motion artifacts were excluded. High-quality images with signal strength index ≥6 were accepted according to the OSCAR-IB criteria (14). Two images from two participants were excluded because of poor quality images [signal strength index (SSI) <6].

The OCT data procurement and description in this study were in alliance with the Advised Protocol for OCT Study Terminology and Elements (APOSTEL) recommendations (15).

### Data and Statistical Analyses

An eye of each patient was included in the data analyses. In occurrences where both eyes met the criteria, the eye was selected according to the higher signal strength index of the macular in the OCT-A imaging; when the SSI of OCT-A images was the same in both eyes, the quality of optic nerve head (ONH) images was decisive. OCT-A and spectral-domain OCT (SD-OCT) results were compared by using multilinear regression, allowing the analysis of the influence of several factors interfering with OCT-A results on a dependent variable. To reduce error variance during the analysis, the total variability of the examined feature was divided into variability caused by the influence of sex, age, eye used (whether left or right eye), hypertension, and SSI.

## Results

Seventy-four participants were enrolled for data analyses. Participants were then divided into two groups according to their Fazekas score (Fazekas scores ≤ 1 and ≥2). Thirty-seven participants, grouped as participants with Fazekas score ≥2 [mean age = 66.19 (7.52) years)], and 37 participants, grouped as participants with Fazekas score ≤ 1 [mean age = 64.91 (4.03) years], were included for data analyses. Significant differences (*P* > 0.05) were not seen in age, body mass index (BMI), and gender. Significant differences were seen in MMSE scores (*P* < 0.001) and Fazekas score (*P* < 0.001) when the two groups were compared as shown in Table 1.

**Table 1:** Demographics and clinical information of participants.

**Parameter**	**Fazekas score ≥2**	**Fazekas score ≤1**	***P*-value**
Number	37	37	
Number of eyes	37	37	
Age (years)	66.19 (7.52)	64.91 (4.03)	0.358
Gender (M/F)	22:15	24:13	
BMI	23.94 (3.50)	22.58 (2.70)	0.812
SBP (mmHg)	135.51 (12.29)	133.41 (14.36)	0.849
DBP (mmHg)	78.75 (9.15)	76.54 (9.02)	0.784
MAP (mmHg)	95.12 (8.72)	94.32 (9.77)	0.910
IOP (mmHg)	12.51 (2.91)	11.64 (3.26)	0.782
BCVA	0.97 (0.20)	1.04 (0.14)	0.071
Fazekas score	3.70 (1.41)	1.0 (0.52)	<0.001
MMSE score	21.09 (3.91)	26.30 (3.13)	<0.001
SSI	7.78 (1.25)	8.08 (1.12)	0.284

*Values are mean (standard deviation). BMI, body mass index; SBP, systolic blood pressure; DBP, diastolic blood pressure; MAP, mean arterial pressure; SE, spherical equivalent; BCVA, best corrected visual acuity; IOP, intraocular pressure; AL, axial length; SSI, signal strength index*.

### Comparison of the Macular Microvascular Density Between the Two Groups

Participants with Fazekas score ≥2 showed significantly reduced RPC density (*P* = 0.02, Table 2) and DCP density (*P* = 0.012, Table 2) when compared with participants with Fazekas score ≤ 1. No significant difference (*P* = 0.584, Table 2) was seen in the SCP when both groups were compared. Additionally, a significant reduction in the peripapillary retinal nerve fiber layer (pRNFL) thickness (*P* = 0.004, Table 2) was seen in participants with Fazekas score ≥2 when compared to participants with Fazekas score ≤ 1.

**Table 2:** Comparison of the macular microvascular densities and structure among the three groups.

	**Fazekas score ≥2**	**Fazekas score ≤1**	***P*-value**
pRNFL (μm)	110.0 (11.09)	116.92 (8.48)	0.004
RPC density, whole (%)	48.45 (3.07)	49.91 (2.11)	0.020
SRCP, whole (%)	45.04 (2.92)	45.41 (2.87)	0.584
DRCP, whole (%)	48.08 (3.46)	50.12 (3.35)	0.012
SSI	7.78 (1.25)	8.08 (1.12)	0.284

*Values were adjusted for age, gender, hypertension, and signal strength index*.

Fazekas scores were significantly associated with the pRNFL thickness (Rho = −0.389, *P* = 0.001), RPC density (Rho = −0.248, *P* = 0.035), and DCP density (Rho = −0.283, *P* = 0.015), respectively, in all participants.

A significant association (Rho = 0.318, *P* = 0.007) was shown between the pRNFL thickness and MMSE scores in all participants. No significant associations (*P* > 0.05) were seen between the microvasculature densities and MMSE scores.

Additionally, RPC density (Rho = 0.439, *P* < 0.0001) and SCP density (Rho = 0.429, *P* < 0.0001) were significantly correlated with pRNFL thickness while adjusting for risk factors.

## Discussion

Our study used the OCT-A technology to assess the retinal capillary densities of the macular and changes around the optic nerve head in healthy participants with white matter hyperintensities. We grouped the participants according to their severity of white matter hyperintensity using the Fazekas scale and analyzed them in order to find distinctive differences between these two groups. We found that participants with Fazekas score ≥2 had significantly reduced DCP when compared to participants with a Fazekas score ≤ 1. Our current study used the OCT-A software to analyze and evaluate the density of the DCP, which is located at the boundary of the deep inner plexiform layer (INL) and outer plexiform layer (OPL). A previous animal study showed that the dominant oxygen consumers of the inner retinal are located in the plexiform layers (16); it has also been reported that these layers are rich in mitochondrial synapses (17). Additionally, pathological reports on brains with WMH have shown mitochondrial dysfunction and high oxygen consumption (18, 19), which reflects that seen in the DCP of the retina. Thus, we suggest that these changes are a reflection of the pathological changes that occur in the brain during the disease cascade of WMH. Second, the deep capillary plexus is aligned along the course of the macular venules and drain into the superficial venules (20) and has been suggested to be involved in the venous side of circulation (21). Previous literature using the fundus camera reported on venular widening in patients with WMH (22–24); Ikram et al. (25) showed that larger venular diameters were associated with the progression of WMH, while De Jong et al. (26) reported that venular widening reflects lower arteriolar oxygen saturation and suggested that venular diameter reflects a lower oxygen supply to the brain. Since changes in the DCP were significantly different among the two groups, our results suggest that the DCP reflects the lower oxygen saturation and also indicates the progression of the disease as previously reported. Additionally, changes in the deep capillary plexus may be as a result of neuroglial loss, which results in impaired interaction between neurons, glial cells, and vascular cells (impaired neurovascular coupling) (27). Neuroimaging reports have shown the gray matter changes, while retinal morphological reports have shown changes in the sublayers of the retina, which constitute the DCP (28) in WMH, indicating the impairment of neurons and glial cells. Since neural activity is associated with the local blood flow (29), alterations in the neuroglial tissue in the inner retina could lead to secondary decrease in the flow density in the capillaries. Thus, in our present study, we suggest that reduced capillary density in the deep capillary plexus may be involved in the pathogenesis of WMH and needs to get more attention.

The layered body of the retinal capillaries is supposed to function as the metabolic support of the different retinal layers. The capillaries of the SCP are engrossed in the ganglion cell layer and the superficial portion of the inner nuclear layer, which contains nuclei of Muller cells, and is devoid of capillaries (30). Moreover, the SCP has been reported to consist of a network of large and small vessels that are connected to the retinal arteries and veins (31). From our OCT-A image, the SCP contains less capillaries when compared to the DCP. Our study did not find any significant difference between the two groups in the SCP; this may be due to the presence of more large vessels in the SCP as compared to the DCP. The transformation of the microvasculature from predominantly capillaries to predominantly small arterioles/venules has a profound effect on oxygen diffusion, thus density. Since majority of oxygen diffusion occur from the capillary vasculature, the presence of fewer capillaries in the SCP may be the reason behind the insignificant difference. Thus, our current report highlights the significance and the role that capillaries play in the pathogenesis and disease cascade of WMH.

Around the optic nerve head is a more superficial capillary network known as the RPCs. The RPCs make up a distinctive vascular network within the RNFL around the optic disk (pRNFL). The RPCs are associated with the highly metabolically active retinal ganglion cell axons, which consists of the RNFL and ganglion cell layer (GCL) (32–34). Due to their parallel structure and rarity of anastomoses, RPCs are suggested to be vulnerable to pathologies, which may affect the retina (35, 36). A previous report (37) evaluated the microvascular density in the macular and around the optic nerve head in migraine patients with white matter hyperintensities; the authors found that the microvascular density around the optic nerve head and densities in the superficial and deep plexus of the macular were significantly reduced when compared to healthy controls. Our current report is quite different from the report aforementioned because our WMH participants were without migraine nor aura but were hypertensive. Nonetheless, we suggest that the OCT-A could be useful to evaluate the macular microvasculature in white matter lesions and could be useful to understand the pathophysiology of this disease.

The decrease in retinal microvascular density in the macular could be secondary to a neurological damage in impaired interaction between the neurons, glial cells, and microvascular cells as reported in the cerebral vasculature (38). Previous reports have shown that retinal alterations in patients with cerebrovascular disorders occur before the detection of retinal vascular alterations suggesting that some neurodegenerative events could precede microvascular changes (39). Due to the association between the neural activity and the local blood flow (40, 41), alterations of thickness of the pRNFL could lead to secondary decrease in capillary flow density.

Our study showed a significant correlation between the pRNFL thickness, RPC network, and DRCP and the severity of white matter lesions using the Fazekas scale, respectively. Fazekas scale is used to quantify the amount of white matter T2 hyperintense lesions. With the retina being a surrogate of the brain, our findings heighten the association of the pRNFL thickness (neuroaxons) and microvasculature in the pathogenesis of WMH. The pRNFL, which has been reported to contain axons, reflect the white matter integrity of the brain (42). Thus, the association between pRNFL thickness and Fazekas score reflect the association between the axonal damage and its severity in the brain. Cerebral microstructural changes in the white matter has been noted for its liability to microvascular damage (43, 44); nonetheless, a weak significant correlation was seen between the microvascular changes of the retina and the Fazekas scores in our current study. Given the association between the retinal microvasculature and cerebral vasculature (45), our study also shows the association between microvasculature damage and its association with the severity of white matter lesions. Although both pRNFL thickness and microvascular (RPC and DRCP densities) damage play significant roles in the pathogenesis of WMH as seen in our data, our data however showed that neurodegeneration is more prevalent than microvascular damage in the white matter damage on MRI using Fazekas scale. Furthermore, the association between the pRNFL thickness and RPC density could suggest that damage of the pRNFL could lead to RPC network damage; this could be translated as neuroaxonal damage leads to microvascular damage in WMH. Studies with larger sample sizes are needed to validate our speculation.

Crucial approaches for diagnosis of cognitive functioning are based on neuropsychological assessment such as MMSE scores (46) and Montreal Cognitive Assessment (MoCA) (47), which are used to evaluate the cognitive status, especially in the aging population. The association between OCT parameters and MMSE can be useful in the clinical valuation and monitoring of patients with cerebral small vessel disease, as the severity of cognitive functioning can be measured with the MMSE score. Our study did not find a correlation between MMSE and the microvascular densities; this is probably because of the lack of psychometrical structure of MMSE, in particular the absence of items reflecting executive functions and psychomotor speed. Additionally, it has been reported (48) that damage related to cerebral small vessel disease is more expressed by MoCA than MMSE and that MoCA is a suited screening tool for patients with cerebral small vessel disease. However, we found a significant association between the MMSE score and the pRNFL, which is congruent with previous reports (49, 50). An association between the pRNFL and MMSE scores suggests that the neurodegeneration associated with the disease cascade causes neuroaxonal damage resulting in a decrease in cognitive functioning.

One limitation of this study is that it was cross-sectional. A longitudinal assessment would be required to validate our hypothesis. Another limitation in this study is that although the current technology of the OCT-A provides the superficial and deep capillary networks of the macular, it is limited by a smaller field of view and that could limit the understanding of the vascular changes in the peripheral retina in WMH patients. Not ending there, despite the presence of the algorithm to reduce motion artifacts, some of the OCT-A images still had motion artifacts at the deep capillary plexus due to the patient's eye movement. The images with artifacts were excluded from the analysis to avoid erroneous results. Further enhancement and refinement of the software are essential to improve its reproducibility and usability for more neurovascular diseases in the future. Additionally, the limitation in our current study was the use of MMSE as the only measure of cognitive impairment. It was used in our current study because it is a standard procedure applied in all hospitals in our Neurology Department to check for cognitive status; second, it is the only cognitive screening tool that is standardized in the Chinese population; and finally, it has been generally used in other studies so we assume that it would provide a much clearer picture of the relation between the microvascular and structural changes in the macular and cognitive impairment in WMH. Another important limitation that should be mentioned concerns projection artifacts caused by superficial vessels projecting shadows onto deeper layers of the retina, which may affect the obtained results. Despite the fact that the latest version of the OCT-A software was used, projection artifacts in the deeper retinal layers were noticeable, which may have the same effect on the results obtained. Furthermore, our sample size was relatively small and may have affected our statistical analyses. Studies with larger sample sizes are needed to validate our hypotheses and delve deeper into the pathophysiological mechanism of WMH.

Despite our limitations, our report showed that microvascular impairment and damage to the neuroaxons are associated with the disease cascade of WMH. We have shown that the RPC and DCP densities are significantly affected, and these impairments are associated with the severity of the disease and cognitive function in MWH. We have shown that OCT-A may be used to study the retinal capillary densities in WMH. Longitudinal studies with larger sample sizes should be performed to evaluate the capillaries in WMH and its risk factors.

## Data Availability Statement

The datasets generated for this study are available on request to the corresponding author.

## Ethics Statement

The studies involving human participants were reviewed and approved by the second affiliated hospital and yuying children's hospital of wenzhou medical university. The patients/participants provided their written informed consent to participate in this study.

## Author Contributions

All authors listed have made a substantial, direct and intellectual contribution to the work, and approved it for publication.

## Conflict of Interest

The authors declare that the research was conducted in the absence of any commercial or financial relationships that could be construed as a potential conflict of interest.
